# Supplementary material for: MetaClass, a Comprehensive Classification System for Predicting the Occurrence of Metabolic Reactions Based on the MetaQSAR Database
Source: Molecules. 2021 Sep 27;26(19):5857. doi: 10.3390/molecules26195857 (PMC8512547; doi:10.3390/molecules26195857)
Supplement: Supplementary file 1 [file molecules-26-05857-s001.zip › Table S2 S3.pdf]

Name	Type of descriptor	Software
Number of Angles<br>Number of Atoms<br>Number of Bonds<br>Number of Torsions<br>Number of Flexible Torsions<br>Number of Heavy Atoms<br>Number of Impropers<br>Number of Rings<br>Number of Chiral Atoms<br>Number of Double Bonds<br>Number of Hbond Acceptor groups<br>Number of Hbond Donor groups<br>Molecular Weight	Constitutive	VEGA
Solvent accessible surface (SAS)<br>Sav<br>Sdiam<br>Molecular VdW Surface<br>Polar surface Area<br>Vdiam<br>Radius of Gyration<br>Molecular Ovality<br>Molecular Volume	Geometrical	VEGA
Lipole<br>Virtual LogP computed by MLP approach	Physicochemical	VEGA
Heat of Formation<br>Total Energy<br>Electronic Energy<br>Core Core Repulsion<br>Molecular Dipole<br>HOMO Energy<br>LUMO Energy<br>Mulliken Electronegativity<br>Parr Pople Absolute Hardness<br>Nucleophilic delocalization (Dn_Total)<br>Electrophilic delocalization (De_Total)<br>self-polarizability (Pis_Total)	Stereo-electronic	MOPAC 2016

Table S2. List of the descriptors included in the study. Details concerning each descriptor can be found in the Manual of VEGA or MOPAC and in the references cited therein.

**Algorithm**	**redox**	**hydrolyses**	**conjugations**	**average**
RF	0.44	0.52	0.36	0.44
ADTree	0.25	0.45	0.33	0.34
BFTree	0.36	0.48	0.31	0.38
ForestPA	0.42	0.50	0.38	0.43
FT	0.38	0.44	0.30	0.37
SimpleCART	0.34	0.50	0.34	0.39
J48	0.40	0.50	0.33	0.41
J48consolidated	0.40	0.49	0.35	0.41
J48 graft	0.40	0.49	0.34	0.41
LADTree	0.26	0.48	0.33	0.36
LMT	0.41	0.52	0.38	0.44
NBTree	0.36	0.43	0.33	0.37
Optimized forest	0.42	0.52	0.35	0.43
REPTree	0.34	0.51	0.31	0.39
SPAARC	0.36	0.51	0.34	0.40
SysFor	0.42	0.48	0.37	0.42
BayesNet	0.24	0.29	0.18	0.24
NaiveBayes	0.21	0.25	0.22	0.23
SVM	0.34	0.10	0.18	0.21
MLP	0.39	0.51	0.36	0.42
SGD	0.33	0.52	0.32	0.39
SMO	0.32	0.51	0.32	0.38
IBK	0.39	0.43	0.30	0.37
Kstar	0.39	0.36	0.28	0.34
Multiclass	0.37	0.47	0.32	0.39
AdaBoostM1	0.27	0.44	0.34	0.35
Regression	0.37	0.47	0.35	0.40
RandomComitee	0.40	0.46	0.34	0.40
Decision Table	0.31	0.49	0.35	0.38
MODLEM	0.39	0.44	0.34	0.39

Table S3. MCC values obtained for the three major classes by using an extended set of classification algorithms implemented by Weka. Details concerning these algorithms can be found within the Weka Manual.
